# The Impact of Heat Waves on Mortality in Seven Major Cities in Korea

**DOI:** 10.1289/ehp.1103759

**Published:** 2012-01-20

**Authors:** Ji-Young Son, Jong-Tae Lee, G. Brooke Anderson, Michelle L. Bell

**Affiliations:** 1 School of Forestry and Environmental Studies, Yale University, New Haven, Connecticut, USA; 2 Department of Environmental Health, College of Health Science, Korea University, Seoul, Korea

**Keywords:** climate change, effect modification, extreme temperature, heat wave, mortality

## Abstract

Background: Understanding the health impacts of heat waves is important, especially given anticipated increases in the frequency, duration, and intensity of heat waves due to climate change.

Objectives: We examined mortality from heat waves in seven major Korean cities for 2000 through 2007 and investigated effect modification by individual characteristics and heat wave characteristics (intensity, duration, and timing in season).

Methods: Heat waves were defined as ≥ 2 consecutive days with daily mean temperature at or above the 98th percentile for the warm season in each city. We compared mortality during heat-wave days and non-heat-wave days using city-specific generalized linear models. We used Bayesian hierarchical models to estimate overall effects within and across all cities. In addition, we estimated effects of heat wave characteristics and effects according to cause of death and examined effect modification by individual characteristics for Seoul.

Results: Overall, total mortality increased 4.1% [95% confidence interval (CI): –6.1%, 15.4%] during heat waves compared with non-heat-wave days, with an 8.4% increase (95% CI: 0.1%, 17.3%) estimated for Seoul. Estimated mortality was higher for heat waves that were more intense, longer, or earlier in summer, although effects were not statistically significant. Estimated risks were higher for women versus men, older versus younger residents, those with no education versus some education, and deaths that occurred out of hospitals in Seoul, although differences among strata of individual characteristics were not statistically significant.

Conclusions: Our findings support evidence of mortality impacts from heat waves and have implications for efforts to reduce the public health burden of heat waves.

Several recent studies have linked heat waves with significant impacts on human health and mortality (Anderson and Bell 2009; D’Ippoliti et al. 2010; Ostro et al. 2009). Understanding the relationship between heat waves and health is crucial given that the frequency, duration, and intensity of heat waves are expected to increase due to climate change. However, the impact of extreme temperatures on mortality has not been characterized in some regions, and few studies have examined the health effects of heat waves in Asia.

Studies on temperature and health have estimated health risks of single days of extreme high temperatures (Curriero et al. 2002; Yu et al. 2010) and have used time-series analysis of temperature modeled as a continuous variable (Pattenden et al. 2003). Other studies have estimated associations between mortality and consecutive days of extreme heat, or heat waves, by comparing outcomes on heat-wave days versus non-heat-wave days (Grize et al. 2005; Naughton et al. 2002). Studies that have estimated the effects of both heat waves and single days of high temperatures have suggested that extended periods of extreme temperatures increase risk beyond that associated with single days of high temperatures (Anderson and Bell 2009; Hajat et al. 2006). However, another recent study in the United States showed that the estimated heat wave effect on mortality was small compared with the main effect of daily high temperature (Gasparrini and Armstrong 2011).

There is no standard definition for heat waves, but most studies have used a combination of temperature (intensity) and duration to define them. In addition to comparing heat-wave days with non-heat-wave days, a few studies have examined the effects of heat wave characteristics, such as intensity, duration, and timing during the year (Anderson and Bell 2009, 2011; Diaz et al. 2002; Hajat et al. 2002, 2006; Kovats and Hajat 2008). For example, a recent study in the United States reported higher mortality risk from heat waves that were more intense or longer (Anderson and Bell 2011), and the U.S. study and a London study both reported stronger associations with heat waves that occurred earlier in the year (Anderson and Bell 2011; Hajat et al. 2002).

Studies have shown that associations between weather and health vary substantially by location (Hajat and Kosatky 2010; Hoffmann et al. 2008); thus, research in different regions is needed. Relatively few studies have investigated the impact of heat waves on mortality in Asia (Huang et al. 2010; Tan et al. 2007). In Korea, associations with single days of high temperature have been investigated (Ha et al. 2011; Kim et al. 2006), but to our knowledge there are no previous studies of heat waves. For the present study, we estimated the effects of heat waves on mortality in seven major cities in Korea during 2000 through 2007 and evaluated effect modification by heat wave characteristics (intensity, duration, and timing in season). We also examined regional differences in effects and effect modification by individual characteristics.

## Materials and Methods

**Figure 1:**
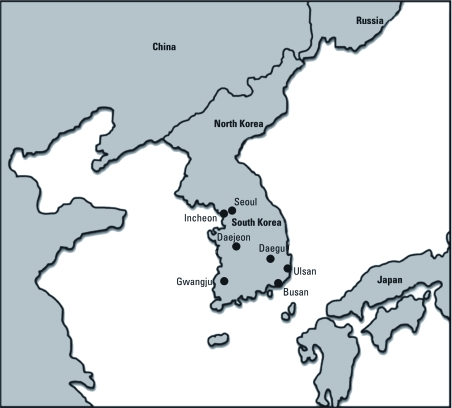
Location of seven major cities in Korea included in this study.

*Data.* We obtained daily counts of deaths for seven major cities in Korea (Figure 1) between 1 January 2000 and 31 December 2007 from the National Statistical Office, Republic of Korea. We considered total mortality defined as all causes of death except external causes [*International Classification of Diseases*, *10th revision* (ICD-10), codes A00–R99; World Health Organization 2007]. The National Meteorological Administration, Republic of Korea provided hourly measurements of ambient temperature and relative humidity for each city that we used to derive 24-hr average values. As in previous studies (Anderson and Bell 2011), we restricted the study period to the warm season (May through September) when heat waves are expected to occur.

*Definition and characterization of heat waves.* We identified heat waves based on a city-specific definition. Specifically, we defined a heat wave as ≥ 2 consecutive days with daily mean temperature at or above the 98th percentile warm season daily mean temperature (for 2000 through 2007) for each city. This definition has been used in previous research (Gasparrini and Armstrong 2011). In addition, we characterized each heat wave by its intensity (average daily mean temperature during the heat wave), duration (length in days), and timing in season [the day in the season when the heat wave occurred (e.g., May 1 = 0, May 2 = 1)]. We also determined whether the heat wave was the first heat wave (first in season) of the year.

*Statistical analysis.* For the first stage, we used city-specific generalized linear models to estimate the difference in total mortality during heat-wave days compared with non-heat-wave days, as described previously (Hajat et al. 2006). We controlled for daily mean temperature, day of the week, relative humidity, and time trends to account for seasonal and long-term trends. The model structure is

ln[*E*(*Y^^c^^_t_*)] = β_0_ + *a^^c^^*HW*^^c^^_t_* + *y^^c^^*DOW*_t_* + ns(Time*_t_*) + ns(*T^^c^^_t_*) + ns(Humidity), [1]

where *E*(*Y^^c^^_t_*) is the expected number of deaths, assumed to follow an overdispersed Poisson distribution for city *c* on day *t*; β_0_ is the model intercept; *a^c^* is the vector of regression coefficients for mortality on heat-wave days versus non-heat-wave days in city *c*; HW*^^c^^_t_* is 0 if day *t* is a non-heat-wave day in city *c*, 1 if day *t* is the first day of any heat wave, 2 if day *t* is the second or later day in the first heat wave of the season, 3 if day *t* is the second or later day in the second heat wave in the season, and so on for HW*^^c^^_t_* values > 3; *y^c^* is the vector of regression coefficients for day of the week for city *c*; DOW*_t_* is the categorical variable for day of the week; ns(Time*_t_*) is the natural cubic spline of a variable representing time to adjust for long-term trends, with three degrees of freedom per warm season (May through September); ns(*T^^c^^_t_*) is the natural cubic spline of temperature for city *c* on day *t*, with three degrees of freedom and equally spaced knots; and ns(Humidity) is the natural cubic spline of humidity for city *c* on day *t*, with four degrees of freedom.

We estimated a separate effect for each heat wave in a city using Equation 1. We then estimated the overall city-specific effect for all heat waves within each city using a city-specific Bayesian hierarchical model, as described previously (Anderson and Bell 2011), and also applied a Bayesian hierarchical model to estimate the overall heat wave effect across the seven Korean cities.

Sensitivity analyses were conducted using different temperature metrics to define heat waves (the minimum and maximum temperature rather than the 24-hr average temperature), and different definitions of heat waves for duration and intensity. We also performed the above analyses using a heat wave definition based on an absolute temperature value rather than city-specific values based on each city’s temperature profile. For this analysis, we used the average of the 98th percentile daily mean temperature for all seven cities instead of the city-specific values.

We estimated how the associations between mortality and heat waves were affected by heat wave intensity, duration, and timing in season using a Bayesian hierarchical model:

^^^^^β*^h^* | α_0_, α_1,_*_j_*, τ^2^ ~ N(α_0_ + α_1,_*_j_*(*X^^h^^_j_* – *x^^–^^_j_*), τ^2^), [2]

where ^^^^^β*^h^* is the estimated association between heatwave *h* and mortality, *X^^h^^_j_* is the heat wave characteristic *j* (intensity, duration, or timing in season) for heat wave *h*; *x^^–^^_j_* is the mean value of characteristic *j* across all heat waves; α_0_ is the ln(relative rate) for average conditions of *x_j_* (when *X^^h^^_j_* = *x^^–^^_j_*); α_1,_*_j_* =is the change in ln(relative rate) for a unit increase in (*X^^h^^_j_* – *x^^–^^_j_*); and τ^2^ is the variance of heat wave effects.

The above model was fitted separately for each heat wave characteristic and city as previously described (Anderson and Bell 2011). All analyses were conducted with packages dlnm and tlnise in R (version 2.10.1; R Foundation for Statistical Computing, Vienna, Austria).

*Further analysis for the city of Seoul.* We performed several analyses restricted to data for Seoul, which has the largest population of any city in Korea. We considered alternative definitions of a heat wave based on six different combinations of intensity (days with daily mean temperatures ≥ 97th, ≥ 98th, or ≥ 99th percentile) and duration (≥ 2 or ≥ 3 consecutive days of high temperatures). We performed additional analysis on timing in season by comparing estimated mortality risks for the first heat wave of the season with later heat waves in Seoul.

We investigated potential confounding by same day ozone and particulate matter (PM) with aerodynamic diameter ≤ 10 μm (PM_10_), because ozone and temperature are often highly correlated, and a previous Korean study indicated higher PM_10_ health effects in the summer (Yi et al. 2010). We obtained hourly ozone and PM_10_ concentrations for Seoul from the Department of Environment, Republic of Korea. We used the maximum daily 8-hr moving average for ozone and 24-hr averages for PM_10_ as the exposure metrics and included ozone and PM_10_ in the model as potential confounders.

In addition, we used stratified models to estimate heat wave effects by cause of death (cardiovascular causes, ICD-10 codes I00–I99; respiratory causes, ICD-10 codes J00–J99) and effect modification by individual characteristics such as sex, age (0–14, 15–64, 65–74, and ≥ 75 years), education level (none, 1–12, and > 12 years), and place of death (out of hospital or in hospital) for Seoul.

## Results

Table 1 shows summary statistics of seven cities and heat wave characteristics in Korea. The mean daily mortality counts ranged from 8.9 in Ulsan to 88.0 in Seoul. Average daily mean temperatures were similar across the seven cities, ranging from 21.9°C in Incheon to 23.3°C in Daegu. For our heat wave definition based on temperatures ≥ 98th percentile and ≥ 2 days duration, the average number of heat waves in the study period was generally similar among cities, ranging from 0.6 to 1.1 per year. Average heat wave intensity (i.e., the average daily mean temperature during heat waves) ranged from 29.0°C in Incheon to 30.7°C in Daegu. Most heat waves lasted 2 or 3 days in all cities, and no heat wave lasted > 5 days. All heat waves were in July or August.

**Table 1:** Summaries of seven cities and heat wave characteristics in Korea, 2000–2007 warm season (May through September).

Daily measures					Heat wave						
City	2007 population	Mortality count (mean ± SD)	Temperature (°C) [mean ± SD (range)]	Relative humidity (%) [mean ± SD (range)]	No. of heat waves per year [mean (range)]	Intensity (°C) [mean (range)]*a*	Duration (days) [mean (range)]	Start date (earliest, latest)
Seoul		10,192,710		88.0 ± 10.2		22.6 ± 3.5 (11.4–30.4)		68.9 ± 13.1 (21.1–96.0)		1.1 (0–3)		29.4 (28.8–30.4)		2.4 (2–4)		July 22, August 17
Busan		3,587,439		42.1 ± 6.9		22.0 ± 3.7 (10.8–30.2)		76.6 ± 11.7 (34.7–99.0)		0.6 (0–3)		29.3 (28.8–30.2)		3.6 (2–5)		July 24, August 5
Incheon		2,664,576		24.8 ± 5.3		21.9 ± 3.5 (11.8–30.9)		75.4 ± 11.8 (26.0–96.9)		0.6 (0–2)		29.0 (28.2–30.9)		3.2 (2–4)		July 26, August 16
Daegu		2,493,261		25.7 ± 5.4		23.3 ± 3.8 (11.2–31.3)		67.3 ± 13.6 (26.5–95.9)		0.8 (0–2)		30.7 (30.4–31.3)		2.7 (2–3)		July 22, August 13
Daejeon		1,475,659		12.7 ± 3.6		22.5 ± 3.5 (11.3–30.0)		71.8 ± 12.1 (28.6–95.8)		0.8 (0–3)		29.1 (28.6–30.0)		2.5 (2–4)		July 22, August 15
Gwangju		1,413,444		13.1 ± 3.6		22.9 ± 3.5 (12.3–30.3)		72.5 ± 11.3 (34.4–96.3)		0.8 (0–2)		29.3 (29.0–29.8)		3.0 (2–5)		July 22, August 14
Ulsan		1,112,799		8.9 ± 3.1		22.5 ± 3.8 (11.1–30.8)		71.3 ± 11.5 (25.0–96.7)		0.9 (0–1)		30.1 (30.0–30.6)		2.4 (2–5)		July 3, August 13
**a**Heat wave intensity measured the average daily mean temperature during heat waves.																

We estimated a significant increase in total mortality in Seoul on heat-wave days compared with non-heat-wave days [8.4%; 95% confidence interval (CI): 0.1%, 17.3% (Table 2)]. The estimated effect was highest in Daegu (9.1%; 95% CI: –12.4%, 36.0%), but the association was not statistically significant. Overall, across the seven cities, we estimated a positive, although nonsignificant, effect of heat-wave days compared with non-heat-wave days on mortality (4.1%; 95% CI: –6.1%, 15.4%).

**Table 2:** Estimated mortality risk on heat-wave days compared with non-heat-wave days based on different heat wave definition by absolute temperature for seven cities in Korea, 2000–2007.

Heat waves defined by relative temperatures: ≥ 2 days with temperatures above the 98th percentile for that city			Heat waves defined by absolute temperature: heat waves defined as ≥ 2 days with temperatures > 29°C		
City	No. of heat waves per year (range)	Effect (%) [mean (95% CI)]	No. of heat waves per year (range)	Effect (%) [mean (95% CI)]
Seoul		9 (0–3)		8.4 (0.1, 17.3)		6 (0–2)		7.8 (–4.3, 21.3)
Busan		5 (0–3)		7.8 (–7.0, 25.0)		4 (0–2)		2.3 (–21.5, 33.5)
Incheon		5 (0–2)		–1.7 (–20.4, 21.3)		3 (0–2)		3.1 (–26.1, 44.0)
Daegu		6 (0–2)		9.1 (–12.4, 36.0)		19 (0–5)		14.9 (7.3, 23.0)
Daejeon		6 (0–3)		–2.3 (–29.6, 35.8)		1 (0–1)		—
Gwangju		6 (0–2)		–9.7 (–29.7, 16.0)		5 (0–1)		–8.1 (–31.8, 24.0)
Ulsan		7 (0–1)		3.1 (–25.9, 43.3)		18 (0–5)		18.9 (3.4, 36.7)
Overall		44		4.1 (–6.1, 15.4)		56		9.9 (–2.2, 23.5)
We estimated a separate effect for each heat wave in a city using Equation 1 and then estimated the overall city-specific effect for all heat waves within each city using a city-specific Bayesian hierarchical model. We also applied a Bayesian hierarchical model to estimate the overall heat wave effect across the seven Korean cities. The city of Daejeon was not included in the analysis using absolute temperature to define heat waves as only one heat wave occurred in that city using that definition.								

We performed several sensitivity analyses using different definitions of a heat wave. Using heat wave definitions based on city-specific minimum and maximum daily temperatures, we estimated overall increases in mortality of 2.3% (95% CI: –8.0%, 13.7%) and 2.3% (95% CI: –7.9%, 13.7%), respectively, on heat-wave days compared with non-heat-wave days. When we defined heat waves as ≥ 2 consecutive days with daily mean temperatures > 29°C, the average of the 98th percentile daily mean temperatures for all seven cities, a total of 56 heat waves occurred in the study period, compared with 44 based on the original definition. With this alternate definition of duration of a heat wave, the overall estimated increase in mortality on heat-wave days compared with non-heat-wave days was 9.9% (95% CI: –2.2%, 23.5%). The effect estimate for Seoul was similar but no longer statistically significant, becoming 7.8% (95% CI: –4.3%, 21.3%) compared with 8.4% (95% CI: 0.1%, 17.3%), possibly because there were fewer heat waves in Seoul when the alternate definition was used (six vs. nine; Table 2). The city of Daejeon was not included in the alternate analysis because only one heat wave occurred based on the alternative definition of a heat wave.

Overall, estimated effects across all seven cities suggest higher mortality risk with heat waves of higher intensity, longer duration, or occurrence earlier in summer, although results were not statistically significant. On average, estimated heat wave mortality risk increased 3.5% (95% CI: –34.5%, 63.6%) for every 1°C increase in average daily mean temperature during heat waves. The central estimate of the association between heat wave duration and mortality was the highest in Daegu, with an estimated increase in mortality risk of 26.2% (95% CI: –25.2%, 112.9%) for a 1-day increase in heat wave duration, whereas the overall estimate across all cities was 2.6% (95% CI: –10.0%, 17.0%). Overall, a 1-day increase in timing in summer, meaning the heat wave began 1 day later in the summer, was associated with 0.2% (95% CI: –1.6%, 1.2%) decrease in the estimated mortality risk during heat waves (Table 3).

**Table 3:** Estimated percentage increase in relative risk of mortality during a heat wave per unit increase in heat wave characteristics, 2000–2007 warm season [estimate (95% CI)].

City	1°C increase in intensity	1-day increase in duration	1 day later in season
Seoul		–2.5 (–26.4, 29.1)		5.7 (–4.9, 17.4)		–0.5 (–1.4, 0.5)
Busan		–14.5 (–52.1, 52.5)		–3.8 (–16.0, 10.2)		–0.6 (–3.6, 2.5)
Incheon		–6.1 (–36.3, 38.4)		–0.9 (–31.2, 42.6)		0.9 (–2.1, 3.9)
Daegu		60.9 (–43.9, 361.2)		26.2 (–25.2, 112.9)		–0.4 (–3.4, 2.7)
Daejeon		29.1 (–74.8, 561.9)		8.2 (–32.3, 72.9)		–1.8 (–5.5, 2.0)
Gwangju		–44.1 (–96.8, 869.3)		–3.7 (–27.4, 27.6)		0.4 (–3.9, 4.9)
Ulsan		118.6 (–53.8, 933.0)		7.3 (–20.5, 44.9)		0.8 (–2.2, 3.9)
Overall		3.5 (–34.5, 63.6)		2.6 (–10.0, 17.0)		–0.2 (–1.6, 1.2)
These heat wave effects by intensity, duration, and timing in season were estimated using the Bayesian hierarchical model given in Equation 2.						

Only one heat wave occurred using the alternative heat wave definition based on ≥ 99th percentile temperature for a duration of ≥ 3 days. With the exception of this case, higher intensity and longer heat waves were associated with higher mortality effects (Table 4).

**Table 4:** Increased risk of mortality for heat-wave days compared with non-heat-wave days under different heat wave definitions in Seoul.

≥ 2 days duration			≥ 3 days duration		
Intensity	No. of heat waves	Estimate (%) [mean (95% CI)]	No. of heat waves	Estimate (%) [mean (95% CI)]
≥ 97th percentile		12		1.8 (–7.2, 11.7)		4		3.8 (–27.1, 47.8)
≥ 98th percentile		9		8.4 (0.1, 17.3)		3		13.5 (–0.1, 28.9)
≥ 99th percentile		2		8.7 (–11.4, 33.4)		1		0.1 (–14.3, 16.9)

As an additional analysis for the timing of the heat wave in the season, we compared mortality risk based on whether a heat wave was the first in its summer in Seoul. The first heat wave of the summer had a larger estimated mortality effect (12.2%; 95% CI: –0.8%, 26.8%) than did later heat waves (2.5%; 95% CI: –16.0%, 25.0%), although the CIs overlapped.

We compared the association between heat waves and mortality with and without adjusting for pollution (ozone or PM_10_) in Seoul. Mean temperature had a correlation of 0.12 with ozone and –0.05 with PM_10_. Estimated heat wave effects were slightly lower after adjusting for ozone (8.0%; 95% CI: –0.3, 16.9%) and for PM_10_ (8.1%; 95% CI: 0, 17.0%).

**Figure 2:**
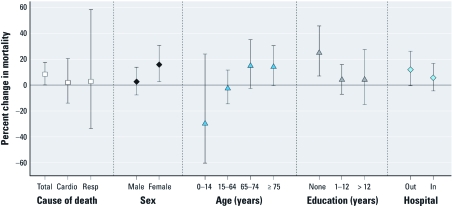
Percentage increase in estimated mortality risk on heat-wave days compared with non-heat-wave days by cause of death and individual characteristics in Seoul. Values are central estimates and 95% CIs. Abbreviations: Cardio, cardiovascular; Resp, respiratory.

Estimated heat wave effects in Seoul were slightly higher for respiratory mortality (2.8%; 95% CI: –33.3%, 58.5%) than for cardiovascular mortality (2.0%; 95% CI: –13.8%, 20.6%), although both of these associations were weaker than they were for total mortality (Figure 2). Estimated heat wave effects were not significantly different by sex, age, education level, or place of death in Seoul (Figure 2) but were higher for females (15.9%; 95% CI: 2.9%, 30.5%) than for males (2.5%; 95% CI: –7.6%, 13.8%), for older residents ≥ 75 years of age (14.8; 95% CI: –2.5%, 35.0%) than for younger residents 65–74 years of age (14.0%; 95% CI: –0.1%, 30.1%), and for those with no education (24.8%; 95% CI: 7.0%, 45.5%) than for those with some education (1–12 years: 4.0%; 95% CI: –6.6%, 15.8%; > 12 years: 4.1%; 95% CI: –14.8%, 27.1%). The estimated heat wave effect for out-of-hospital deaths (11.9%; 95% CI: –0.6%, 26.0%) was higher than for in-hospital deaths (5.5%; 95% CI: –4.4%, 16.4%).

## Discussion

In this study, we analyzed the association between heat waves and mortality considering heat wave characteristics (intensity, duration, and timing in season) in seven major cities in Korea. We also examined regional differences in effect estimates by estimating effects for different cities and investigated effect modification by individual characteristics for Seoul. The overall estimate across the seven cities indicated a positive association between mortality and heat-wave days compared with non-heat-wave days, although estimates varied among the cities, and only the estimate for Seoul was positive and statistically significant. The estimated impact of heat waves on mortality was higher with higher intensity, longer duration, or earlier occurrence in summer; however, the effects were not statistically significant. We also found some evidence of increased susceptibility in women, the elderly, and residents with no education, and stronger associations with deaths occurring outside of a hospital compared with deaths in a hospital for Seoul.

In this study, the city-specific effect of heat waves on total mortality ranged from –9.7% to 9.1%. The highest central effect estimate was observed in Daegu with higher temperatures, whereas some cities did not have a positive effect. A recent study by Anderson and Bell (2011) estimated mortality risk for heat waves (defined as ≥ 2 consecutive days with daily mean temperature ≥ 95th percentile for the community) in 43 U.S. cities and reported heterogeneity between communities in heat wave effects. Specifically, estimated heat wave mortality impacts were more pronounced in the Northeast and Midwest than in the South, which the authors hypothesized was due to differences in physical acclimatization, levels of exposure, community-level responses to heat waves, and other physiological and behavioral characteristics of the populations and communities. A study of nine European cities reported heterogeneity among city-specific estimates of the impact of heat waves on mortality, with the highest estimated impact in Mediterranean cities that experience more frequent and higher temperature heat waves than did cities in northern Europe (D’Ippoliti et al. 2010).

Our results suggest that the intensity, duration, and timing of heat waves may influence the risk of mortality, although these findings were not statistically significant. Estimated mortality risk increased with intensity or duration of heat waves in a U.S. study (Anderson and Bell 2009), and another study estimated higher mortality effects for longer heat waves (Diaz et al. 2002). Our findings suggest that heat waves earlier in the summer were associated with higher mortality than were later heat waves and that the first heat wave of the summer had a greater impact than did later heat waves, consistent with studies by Smoyer (1998) and Anderson and Bell (2011). Previous studies suggest that increased risks associated with earlier heat waves could be due to several factors, including mortality displacement and adaptive behavior (Braga et al. 2001; Le Tertre et al. 2006; Nitschke et al. 2007).

We estimated a stronger effect of heat waves on respiratory mortality than cardiovascular mortality, consistent with other studies. Hoffmann et al. (2008) reported that the effect of heat waves was more prominent for respiratory mortality than cardiovascular mortality in Essen, Germany. Another European study also estimated stronger effects of heat waves on respiratory mortality than cardiovascular mortality in most cities, which the authors suggested may reflect increased susceptibility among people with preexisting chronic respiratory diseases (D’Ippoliti et al. 2010). However, the association between heat waves and total mortality was stronger than associations with either respiratory or cardiovascular mortality among Seoul residents in our study.

Mechanisms that determine mortality risk may differ between heat waves and single days of high temperatures. For example, the lack of nightly cooling and longer duration of heat may contribute to increased mortality during heat waves (Hoffmann et al. 2008). Prolonged exposure to extreme heat might induce hyperventilation, leading to dyspnea, dehydration, and mechanical and cardiovascular effects (Schifano et al. 2009), and physical problems related to heat, including dehydration (Worfolk 2000), could worsen over the course of a heat wave. Additionally, heat may accumulate in buildings without air conditioning during a heat wave (Henschel et al. 1969), particularly if nighttime temperatures remain high or windows are not opened for ventilation (Givoni 1998), and in large, poorly ventilated residential buildings in urban areas (Clarke 1972).

In this study, we estimated stronger associations among females than males, although estimates were not significantly different. Previous studies also reported differences by sex (D’Ippoliti et al. 2010) that may be attributable to factors such as physiological differences between males and females (e.g., the ability to regulate body temperatures to heat stress) or confounding by age and social conditions (e.g., living alone, income level) (Barnett 2007; D’Ippoliti et al. 2010). On the other hand, other studies have reported no differences in estimated heat wave effects on mortality by sex (Basu and Ostro 2008; Huang et al. 2010; O’Neill et al. 2003). The nature of susceptibility in heat wave–mortality relationships is complex and may be related to factors such as population demographics, socioeconomic status, housing characteristics, and adaptation to local climate. For example, women in our Seoul study population were older and less educated than were males.

Consistent with a few previous studies (Huang et al. 2010; McGeehin and Mirabelli 2001; Schifano et al. 2009), our results suggest that the elderly were more susceptible to heat waves. This may reflect factors such as impaired physiological responses to heat stress (e.g., elevated sweating thresholds, decreased skin blood flow, reduced cardiac output) and preexisting chronic diseases for the elderly (Foster et al. 1976; Kenney and Munce 2003). In addition, older people are more likely to live alone and be socially isolated (Hajat and Kosatky 2010; Klinenberg 2002).

We found some evidence that Seoul residents with no education were particularly vulnerable to heat wave mortality. Education may be an indicator of low socioeconomic status, which could be related to poor baseline health status, limited access to health care, and housing conditions such as the lack of air conditioning and electric fans (McGeehin and Mirabelli 2001). Previous studies have also reported that those with low education and socioeconomic status had greater susceptibility to heat-related mortality (Borrell et al. 2006; O’Neill et al. 2003).

Our findings suggest that heat waves had a higher mortality effect for those dying outside a hospital than those dying inside a hospital. A previous study reported that those dying outside a hospital were more susceptible to extreme temperatures, especially to heat, compared with deaths inside a hospital (Medina-Ramon et al. 2006). These findings support the hypothesis that exposure to extreme ambient temperature affects mortality (e.g., air filtration and air conditioned or heated environment in a hospital vs. outside a hospital). On the other hand, location of death in a hospital may also reflect other factors such as whether the decedent had health insurance and access to health care (O’Neill et al. 2003).

Our analysis was based on a relatively small number of heat waves and deaths compared with other studies, and we were not able to account for many potential confounding factors, such as levels of chronic disease. In addition, the exact specification of a heat wave, and thereby estimates of heat-wave–related mortality, vary among studies, and there are no standard criteria or models. Further study is needed to address these issues.

Efforts have been made to reduce heat-related impacts on human health in Korea, including implementation of heat health warning systems based on Kalkstein spatial synoptic classification as well as heat alert systems based on heat index (National Institute of Meteorological Research 2011; Korea Meteorological Administration 2011). However, public health interventions need scientific evidence to effectively target at-risk groups. Korea is rapidly becoming an aging society, with more persons who are socially isolated, elderly, and/or living alone. These groups may be particularly susceptible to heat related mortality.

To the best of our knowledge, this is the first study in Korea to investigate the impact of heat waves on mortality. Further, it is the first study for Korea to examine heat wave effects on mortality by heat wave characteristics and the first to examine how heat wave effects may be modified by individual characteristics. Considering that heat waves are expected to be more frequent, longer, and more intense in the future due to climate change, increased understanding of how heat waves affect health is needed. Our findings provide supportive evidence of the impact of heat waves on mortality and have implications for policy makers to reduce the burden of heat wave mortality.
